# Mathematical simulation of damage detection for fighting athletes and equipment based on conjugated polymer development

**DOI:** 10.3389/fchem.2023.1286290

**Published:** 2024-01-08

**Authors:** Yang Lu, Yang Zhao, Jingyun Wu, Xiaoyan Chen, Qijia Zhang

**Affiliations:** ^1^ Center for Physical Education, Southern University of Science and Technology, Shenzhen, Guangdong, China; ^2^ Department of Physical Education, Sun Yat-Sen University, Guangzhou, Guangdong, China; ^3^ Martial Arts Academy, Guangzhou Sport University, Guangzhou, Guangdong, China; ^4^ The Education Department, Liaoning Special Education Teachers College, Shenyang, Liaoning, China

**Keywords:** sports injury, conjugated polymer, polypropylene material, fighting athlete, mathematical simulation

## Abstract

Traditional combat sports equipment usually uses synthetic materials, such as polyurethane and synthetic leather. Although these materials have a certain degree of strength and durability, they have poor flexibility and antibacterial properties, making it difficult to provide stable support and protection for athletes. In order to enhance the antibacterial properties and flexibility of sports equipment and reduce the risk of injuries to athletes, this article conducts in-depth research on the development of combat sports equipment using conjugated polymers. This article first selects polypropylene (PP) as the base material for sports equipment for combat athletes, and uses the gas phase polymerization method to prepare the material; then uses chitosan as an antimicrobial agent and uses the oxidative degradation method to prepare it; after that, this article coats the chitosan antibacterial agent on the prepared PP material, and uses a combination of dipping and calendering for antibacterial treatment; finally, this article uses the spunbond melt-blown composite method to fill and combine the top equipment of combat athletes to achieve the structural design of sports equipment. In order to verify the effectiveness of the equipment, this article conducted equipment performance testing and sports injury simulation. The results showed that the average diameter of the antibacterial zone of this sports equipment reached more than 1 mm, and in the injury risk test, the risk of athletes' joint and muscle injuries was reduced by 16.9% and 20.5% respectively. Research shows that developing combat sports equipment based on conjugated polymers can help reduce the risk of injury to athletes and improve the safety of combat sports.

## 1 Introduction

With the enrichment of the material and spiritual life of the masses, fighting sports have achieved great development in the sports market. As a highly adversarial competitive sport, fighting athletes often experience training injuries during training and competition. Traditional equipment materials have limitations such as poor antibacterial effects and low flexibility, making it difficult to ensure the safety of athletes. Improving equipment performance and developing sports equipment with high resistance and flexibility are of great significance for improving the functionality and safety of fighting sports competitions. With the development and progress of organic chemistry, the application of conjugated polymer materials in professional fields such as organic electronics and biomedicine is becoming increasingly widespread. Conjugated polymers have a low density and can achieve lightweight design while ensuring equipment strength, helping to reduce the burden on athletes and improve wearing comfort. In addition, it has good flexibility and bending performance, which can produce soft protective gear and equipment, helping to provide better protection and flexibility. Polypropylene, as a conjugated polymer composed of single or multiple structural units, has characteristics such as antibacterial, lightweight, and strong mechanical properties. In fighting sports, incorporating conjugated polymers into the design and development of sports equipment has important practical value for effectively improving the antibacterial effect, flexibility, and mechanical properties of sports equipment, and reducing the risk of sports injuries for athletes during training and competition.

Sports equipment plays a crucial role in ensuring the safety of daily sports or training. To improve the tensile strength of equipment, Panchagnula Kishore Kumar improved the performance of glass fiber reinforced polymer materials by filling a small amount of multi walled carbon nanotubes. The bending test showed that multi walled carbon nanotubes were effective in improving the tensile strength and other mechanical properties of sports equipment (Panchagnula and Kuppan, 2019). In order to change the material structure and improve its tensile strength performance, Sabahi Namini Abbas used spark plasma sintering technology to analyze the effect of adding boron carbide and titanium diboride on the microstructure development and mechanical properties of equipment materials. It was found that the elongation and ultimate tensile strength of equipment samples doped with boron carbide or titanium diboride were higher than those of single titanium equipment samples (Namini et al., 2018). To improve the impact resistance of sports materials, Duncan Olly reviewed the application and design of expansion materials in sports protective equipment, and introduced common expansion materials, structures, and their advantages in reducing sports injuries (Duncan et al., 2018). To improve the mechanical properties of sports equipment, Reich Matthew J used low melting point thermoplastic rapid prototyping technology to manufacture recycled polycarbonate particle equipment parts, and compared them with silk screen printing and bulk raw materials. The results indicated that compared to the other two materials, equipment parts made from recycled polycarbonate particles had stronger tensile strength (Reich et al., 2019). In order to achieve high mechanical properties of materials, Rajak Dipen Kumar implemented a new efficient manufacturing process by combining synthetic or natural materials, using natural materials as components in the combination of matrix materials and reinforcement materials, to obtain equipment materials with high mechanical properties and degradability (Rajak et al., 2019). In order to achieve better mechanical properties and adhesion for sports equipment, Dilfi KF Anna used hemp strands as natural lignocellulose fibers and obtained improved polypropylene composite materials through injection molding. He verified its good mechanical properties through experiments (Dilfi et al., 2018). Currently, the development of sports equipment has made good progress. However, with the professionalization of fighting sports, appropriate improvements and optimizations need to be made to sports equipment materials, and current research has not fully considered the flexibility of sports equipment.

Conjugated polymers have a lower density and can effectively enhance the flexibility of materials. In order to prepare more flexible equipment materials, Hu Xiaoyong improved the original material by using a star shaped cage shaped sesquioxane conjugated polymer with intramolecular heterogeneity. This star shaped hybrid conjugated polymer has higher flexibility performance compared to its linear analogues, and its conjugated stacking effect greatly increases the adhesion force between the polymer and carbon tubes, which is beneficial for improving the flexibility of equipment materials (Hu et al., 2020). To reduce material density, Qu Muge used conjugated conductive polymers as functional materials and nanocellulose as matrix materials to prepare conjugated conductive polymer nanocellulose composite materials. It combined the excellent flexibility of conjugated conductive polymers and the degradability of nanocellulose, expanding the range of material development and application (Qu et al., 2019). To improve material flexibility, Zhang Huijun introduced heteroatoms into the conjugated nanoring spectra and redox reactions, causing changes in assembly behavior by changing the structure, thereby bringing more flexible performance to conjugated materials and providing new elements for the controllable construction of multifunctional supramolecular systems (Zhang and Lin, 2022). Conjugated polymers can fully enhance the flexibility of materials. However, most studies have not provided more effective guidance for the development of equipment antibacterial effects by combining practical issues related to fighting sports injuries, and only explored the application of conjugated polymers in material properties improvement from a theoretical perspective.

**FIGURE 1:**
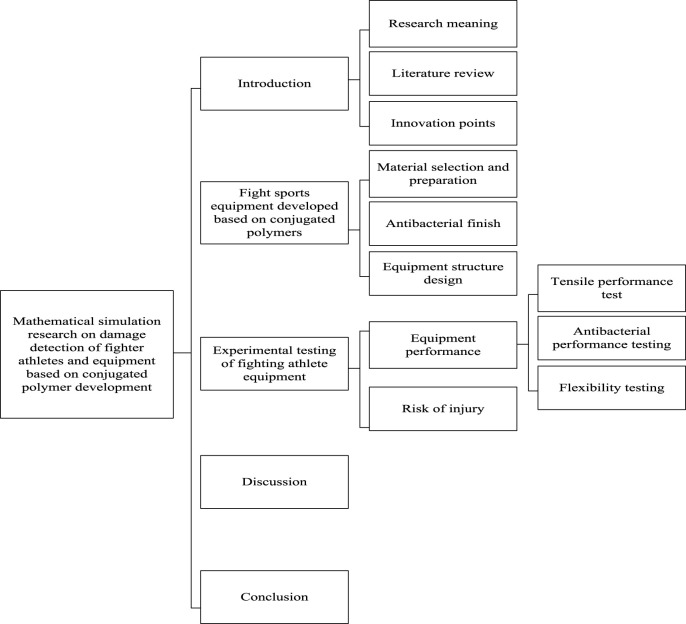
Research framework of this article.

In order to improve the performance of fighting sports equipment and reduce the risk of athlete injury, this article conducted in-depth research on the development of fighting sports equipment for athletes using conjugated polymers. The effectiveness of the equipment was verified through equipment performance testing and mathematical simulation of sports injury. Compared to traditional polyester fiber equipment materials, the sports equipment developed based on PP material in this article had significant advantages in terms of tensile performance, with a maximum tensile strength increase of about 31.7%. In addition, the equipment developed in this article had ideal antibacterial performance and flexibility. In antibacterial testing, the maximum diameter of the antibacterial ring of the material in this article reached 1.75 mm, which could effectively inhibit the growth of bacteria in sports environments and avoid athlete injury and infection. Moreover, the quality of the sports equipment developed in this article was more than 130 g lighter than traditional polyester fiber materials. Traditional research on damage detection for combat athletes and equipment mainly focuses on material mechanical properties, biomechanical properties, sensor technology, etc. There is a certain gap in material innovation and damage detection. The development and application of conjugated polymer materials in this article bring new ideas and possibilities for the research of combat sports equipment. In the simulation of fighting sports injuries, this equipment can effectively reduce the injury risk of athletes and provide safety guarantees. The research framework of this article is shown in Figure 1.

## 2 Fighting sports equipment developed based on conjugated polymers

### 2.1 Selection and preparation of conjugated polymer materials

Fighting sport is a comprehensive and adversarial sport characterized by practical combat (Podrigalo et al., 2018; Ouergui et al., 2021). As a high-intensity sports event, in order to prevent athletes from sports injuries to a certain extent, the development and design of fighting sports equipment not only focuses on the mechanical properties of materials, but also has strict requirements for the flexibility and antibacterial performance of materials.

Polypropylene belongs to linear olefin conjugated polymers, with overall properties similar to polyethylene, but with fewer unsaturated double bonds and an additional methyl group as the side chain compared to polyethylene. Therefore, in addition to its superior performance in thermal and mechanical properties, it also has very stable physical and chemical properties (Cheng et al., 2018; Park and Lee, 2021). In practical applications, it also exhibits excellent anti fatigue, antibacterial and other performance characteristics. Therefore, this article chooses polypropylene as the matrix material for fighting athletes’ sports equipment. The main reagents and raw materials used in the preparation of polypropylene are shown in Table 1, and the main equipment used is shown in Table 2.

**TABLE 1:** Main reagents and raw materials.

Sequence	Reagents and raw materials	Purity
1	Propylene	99.9%
2	Ethylene	99.9%
3	Hydrogen	99.9%
4	Nitrogen	99.9%
5	Catalyzer	-
6	N-Heptane	99%
7	Anhydrous ethanol	99.5%
8	Xylene	Analytical Reagent (AR)
9	Isopropanol	AR
10	Hydrochloric acid	AR
11	Sodium chloride	AR
12	Potassium hydroxide	AR

**TABLE 2:** Main equipment for polypropylene preparation.

Sequence	Device	Model
1	High temperature reaction kettle	YZ-100KJ
2	Buffer tank	M250049
3	Magnetic stirrer	GL-5250C
4	External circulation water area	DL-1005

In Table 2, the main equipment for polypropylene preparation includes high-temperature reaction kettle, buffer tank, magnetic stirrer, and external circulating water area.

Firstly, the gases used in Table 1 are purified by treating the higher amounts of water and oxygen in the gases such as propylene, ethylene, nitrogen, and hydrogen to the required levels for the synthesis reaction. The gas required for the preparation process is discharged using a steel cylinder and introduced into two purification tubes containing molecular sieves.

Then, the solvent used in the preparation process is pre-treated and the impurities contained in the solvent are removed by extraction. The specific steps are as follows: a certain amount of potassium hydroxide is added to the solvent and dried under closed conditions for 5 days. Then, the water is completely removed and added to the solvent purification equipment. After cleaning with nitrogen for 2 h, it is then loaded into the solvent storage tank. Solvent purification is the process of using nitrogen gas to extrude the solvent from the solvent storage tank under a certain pressure, and then sequentially entering purification pipelines containing reactive metals such as aluminum and copper, completing the process of deoxygenation and dehydration. After treatment with solvent purification equipment, the content of water and oxygen impurities is purified to below the standard range of 5 ppm (parts per million).

**FIGURE 2:**
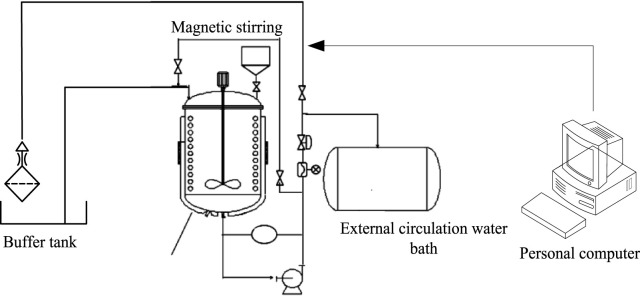
Reaction device architecture.

As shown in Figure 2, the pre treated solvent is fed into a reaction device composed of a buffer tank, a magnetic stirrer, an external circulating water area, and a personal computer, and a catalyst is added. Among them, the function of the buffer tank is to stabilize the pressure fluctuation of the entire reaction device and supply the mixer required for the entire reaction process; the magnetic stirrer mainly assists in achieving sufficient gas reaction and ensuring the sealing of the reaction process; the external circulation water mainly provides heat for the entire reaction device; personal computers are mainly used to control and present parameters such as temperature, pressure, and speed during the reaction process.

Using sodium chloride as the main raw material, steps such as polymerization and baking are used for material polymerization. Firstly, the external circulation water area is adjusted to control the temperature of the entire reaction device at 500°C. Sodium chloride crystals are baked under these environmental conditions for 150 min, and then the roasted sodium chloride is poured into the reactor for another 70 min of baking with salt. The water bath temperature is adjusted to the specified temperature, and the catalyst and internal electron donors are added to the reaction device through n-heptane. Then, stirring is started to discharge the added n-heptane solvent. Under specified conditions, a certain amount of hydrogen is injected inward by opening the hydrogen pipeline valve, and a certain amount of ethylene is injected outward by opening the ethylene pipeline valve. Finally, the propylene pipeline valve is fully opened to continue outward transportation, thus achieving gas-phase copolymerization. After the polymerization is completed, the delivery of propylene is stopped, restoring the pressure in the reactor to normal pressure. Finally, at a temperature of 500°C, the acidified ethanol is used to clean, filter, and dry the polymer under vacuum conditions for 10 h, ultimately obtaining the PP material.

### 2.2 Antibacterial finishing

Untreated PP materials have insufficient resistance to liquid contamination and require antibacterial finishing to achieve better protective effects. The bacterial proliferation of liquid pollutants on the surface of polymer materials undergoes three stages: contact, infiltration, and diffusion, and these three stages interact and influence each other (Luo et al., 2021). When a solid comes into contact with a liquid in air, the interface between gas and solid becomes the interface between solid and liquid. The solid-liquid interface increases with the increase of infiltration degree. The diffusion state refers to the state where the adsorption work of a fluid on a solid has exceeded its own surface tension. Using the contact angle as an indicator, the force state of the droplet on the solid surface is obtained, which is calculated using the following formula (Eq. 1) (Liu et al., 2020):

$$T_{l}-T_{g}+T_{\mathrm{lg}}\cos\theta =0$$ (1)

The definition of formula variables is shown in Table 3.

**TABLE 3:** Definition of formula variables.

Sequence	Variable	Meaning
1	$T_{l}$	Surface tension of liquids and solids
2	$T_{g}$	Surface tension of gas and solid
3	$T_{\mathrm{lg}}$	Surface tension of liquids and gases

By measuring and analyzing the contact angle, suitable materials can be selected, designed, or surface modified to improve the antibacterial performance of combat equipment. This helps to reduce the breeding of bacteria, reduce the risk of infection, and protect the health of athletes. If θ=0 , it indicates that liquid pollutants have completely infiltrated the material surface and have undergone a certain degree of diffusion. If θ=90° , it indicates that liquid pollutants have partially infiltrated the surface of the material. If θ>90° , it indicates that the material has a certain degree of hydrophobicity. If θ=180° , it indicates that the material has complete hydrophobicity. However, in practical applications, the situation of θ=180° does not exist.

In order to achieve better protective effect of PP material, this article uses antibacterial agents to carry out antibacterial finishing on it. Chitosan is a highly bioactive natural macromolecule with good biocompatibility and biodegradability (Wang et al., 2018). It utilizes positively charged ammonium ions formed by amino acids in acidic solutions to interact with negatively charged cell surfaces, and then achieves antibacterial effects through comprehensive chemical reactions such as chelation, electrostatic attraction, and repulsion of multidentate ligands (Cavallaro et al., 2021). This article uses chitosan as an antibacterial agent and uses oxidative degradation method to prepare the antibacterial agent. The reagents and equipment used for preparation are shown in Tables 4, 5.

**TABLE 4:** Reagents used for preparation.

Sequence	Reagent	Purity
1	Trimethylammonium chloride	99%
2	Acetone	AR
3	Chitosan oligosaccharide	AR
4	Anhydrous ethanol	99.5%
5	Deuterium depleted water	120 ppm
6	potassium bromide	Spectrography

**TABLE 5:** Equipment used for preparation.

Sequence	Device	Model
1	Electronic balance	FA 2004N
2	Mixer	AE300L-P
3	Vacuum oven	DZF6020
4	Centrifuge	Centrifuge 5418R
5	Fourier Transform Infrared Spectrometer	YSM-8102-01
6	Biochemical incubator	HD-E702
7	Thermostatic magnetic stirring pot	DU-3GO

Firstly, the oligomers in the raw chitosan oligosaccharide are purified, and the oligomer powder is dissolved in deionized water. Then, it is filtered with a 300 mesh nylon filter yarn to remove insoluble impurities and corresponding enzymes. After frequent room temperature drying, pure chitosan oligosaccharide materials can be obtained.

**FIGURE 3:**
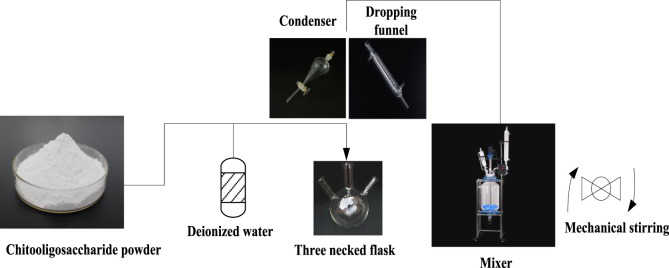
Mixing of chitosan oligosaccharide powder.

As shown in Figure 3, the obtained pure chitosan oligosaccharide material is placed in a three necked flask, and 5.3% (Mass/Volume) of deionized water is added. A condenser tube and a drip funnel are installed on the flask, and they are placed in a stirring pot and mechanically stirred at a rate of 500 revolutions per minute. The temperature is gradually raised to 55°C, and in this case, the chitosan oligosaccharides are continuously stirred to disperse.

A pipette is used to absorb a certain amount of trimethyl chloride and place it in deionized water. It is thoroughly stirred and dissolved, and then poured into a constant pressure drip funnel. The drip rate is controlled to ensure that the mixed solution is dropped within 45 min. The final volume of deionized water during this process should reach a concentration of 0.06 g per milliliter with chitosan. At a temperature of 55°C, all materials are added to the solution, and the solution is then reacted at a temperature of 80°C. Chemicals with lower purity and more impurities separated from the reaction system are injected into a centrifuge tube and the product is precipitated using acetone. At a temperature of 50°C, the product is dried. In order to obtain a purer product, the product is dialyzed in deionized water for 3 days and dried in a dryer for 36 h to obtain an antibacterial agent.

**FIGURE 4:**
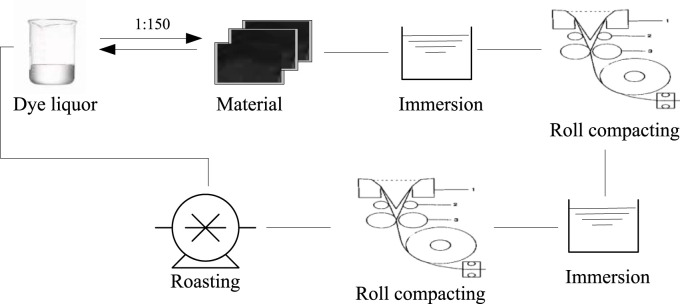
Antibacterial finishing of materials.

In order to enable the antibacterial agent to exist on the surface of the material in a physical adsorption or chemical grafting state, this article arranges the antibacterial agent on the prepared PP material and uses a impregnation rolling combination method for post-treatment, as shown in Figure 4.

Due to the need to ensure good flexibility in fighting sports equipment, in order to achieve a more uniform and stable mechanical and antibacterial effect of the material, the ratio of the dye solution prepared during impregnation to the material quality is chosen to be one to one hundred and fifty. After soaking for 60 min, it is then subjected to one more press, and this process is repeated 1–2 times. After completing this step, it is then baked, at which point the solution mass in the material reaches about four-fifths of the total mass of the material. The pre baking temperature is set to 55°C. Firstly, it is pre baked for 10 min to ensure a constant temperature. Then, under constant temperature conditions, the material is baked for 5 min to obtain a dried PP material sample after antibacterial treatment.

### 2.3 Structural design of equipment

**FIGURE 5:**
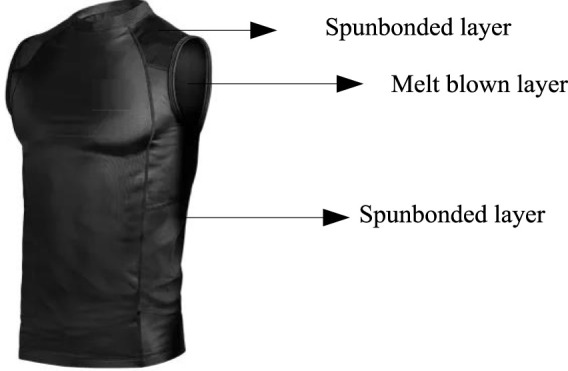
Top equipment level.

In the structural design of equipment, this article uses antibacterial treated PP material samples as raw materials to design the structure of fighting athlete’s top equipment. Spunbonded melt blown composite method is a commonly used textile material preparation process, usually used to prepare fiber materials or non-woven fabrics. This method combines the characteristics of spunbond method and melt blown method, which can to some extent overcome their respective shortcomings and achieve comprehensive utilization of material composition and functionality. Due to the inability of PP material to be sewn through a sewing machine, this article uses the spunbond melt blown composite method to fill and combine the top equipment of fighting athletes. The top equipment structure is divided into three parts: spunbond layer, melt blown layer, and spunbond layer, as shown in Figure 5.

In Figure 5, the spunbonded layer mainly provides mechanical properties, which have excellent strength and wear resistance, and the melt blown layer has excellent filtration, blocking, and antibacterial performance. In practice, the quality, strength, and filtration performance of equipment can be adjusted by adjusting the melt blown layer according to the competition and training needs of athletes. According to the hierarchy of top equipment, firstly, spunbond method and melt blown method are used to construct PP material fiber mesh. Then, according to the structure, PP material fiber mesh is combined. Finally, by hot rolling bonding and overlapping with each other, a whole shape is formed.

In the process of implementing local development of top equipment, PP material is used for local filling. In this process, due to the large number of inflection points, curvature, and unevenness of the human body model itself, the PP material is cut into designed shapes, divided into different shapes and sizes, and then bonded to the model through shards to achieve seamless connection. Finally, by using methods such as bonding and melt spraying, the PP material is bonded to the 3D (three-dimensional) model, in order to achieve precision and aesthetics as much as possible while satisfying the shape requirements. Then, it is integrated with the clothes to form a more complete equipment form.

## 3 Experimental testing of fighting athlete equipment

To verify the effectiveness of fighting sports equipment developed based on conjugated polymers, this article conducted experimental analysis from two perspectives: equipment performance and injury risk. Among them, equipment performance mainly evaluated the tensile performance, antibacterial performance, and flexibility of fighting sports equipment developed with conjugated polymers. The risk of injury was evaluated by simulating the movement status of fighting athletes while wearing equipment.

### 3.1 Equipment performance

To highlight the performance and effectiveness of equipment, this article compared the fighting sports equipment developed by conjugated polymers with traditional polyester fiber fighting sports equipment on the market, and evaluated them from three aspects: tensile performance, antibacterial performance, and flexibility.

(1) Tensile performance

Fighting sports equipment has strict requirements for material tensile performance. Generally speaking, the higher the tensile strength and elongation at break of a material, the stronger its resistance to external force damage. The stretching performance of fighting sports equipment plays an important role in ensuring the safety of athletes. This article used a tensile testing machine to test the tensile performance of fighting sports equipment based on PP material. At room temperature, each group tested 10 material samples, and the average of the 10 test results was taken. The model and tensile parameter settings of the testing machine are shown in Table 6.

**TABLE 6:** Testing machine model and tensile parameter settings.

Sequence	Item	Specifications
1	Testing machine model	XLW-H
2	Force value	30N(Newton)
3	Accuracy	0.5
4	Stretching speed	15 mm per second
5	Stretch size	60 mm × 20 mm × 5 mm

**FIGURE 6:**
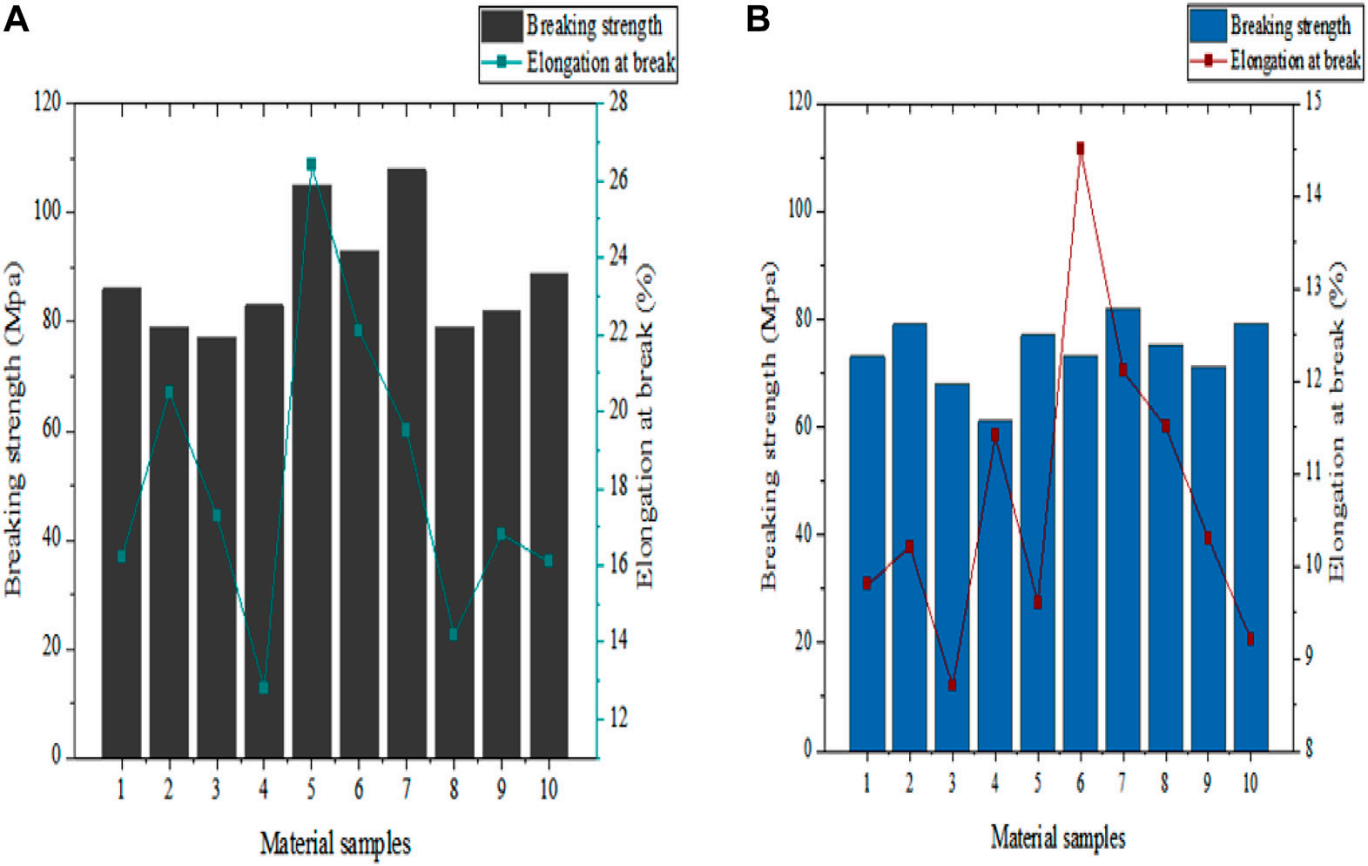
Tensile performance test results. **(A)** shows the tensile performance of PP material in this article. **(B)** shows the tensile performance of polyester fiber materials.

In Table 6, the stretching speed for the tensile strength experiment in this article was set to 15 mm per second, and the stretching size was set to 60 mm × 20 mm × 5 mm. The test results are shown in Figure 6.

The material tensile strength of combat equipment generally needs to be in the range of 60 MPa–120 MPa. This range can provide sufficient strength and durability to ensure that the equipment can withstand a certain amount of impact and stretching force during combat sports. In Figure 6, the horizontal axis represents the material samples used for testing, while the vertical axis represents the tensile strength and elongation at break of the material. From the overall test results, it can be seen that the sports equipment material developed based on conjugated polymers in this article had superior tensile performance. In Figure 6A, the maximum tensile strength of the equipment material in this article reached 108 MPa (Mega Pascal), and its maximum fracture elongation rate reached 26.4%. In Figure 6B, the maximum tensile strength of polyester fiber material reached 82MPa, and its maximum breaking elongation rate reached 14.5%. From the comparison of tensile performance, it can be seen that compared to the highest tensile strength of traditional polyester fiber materials on the market, the highest tensile strength of PP material in this article increased by about 31.7%. There is a conjugated π electron system in the molecular chains of conjugated polymers, which makes the molecular chains highly rigid and stable. The saturated bonds in polypropylene materials also make them have good chemical and heat resistance, thereby improving the mechanical properties of sports equipment.

(2) Antibacterial performance

In fighting sports, the moisture and nutrients in athletes’ sweat can provide a favorable environment for bacterial growth. If the equipment cannot effectively inhibit bacterial growth, the risk of injury and infection for athletes is increased. In antibacterial testing, this article used agar plate diffusion method to evaluate the antibacterial performance of two major equipment materials against *Escherichia coli* and *Staphylococcus aureus*. Firstly, bacterial strains were cultured until the logarithmic growth phase, and then sterile physiological saline was used to dilute to an appropriate concentration to obtain bacterial suspension. The bacterial suspension was evenly coated on the surface of the agar plate. The parameter settings for bacterial culture are shown in Table 7.

**TABLE 7:** Setting of bacterial culture parameters.

Sequence	Item	Parameter specifications
1	Cultivation temperature	35°C–37°C
2	Cultivation time	4–8 h
3	Bacterial liquid concentration	1.5 × 108 Colony forming units per milliliter
4	Extraction volume	10 mL

**FIGURE 7:**
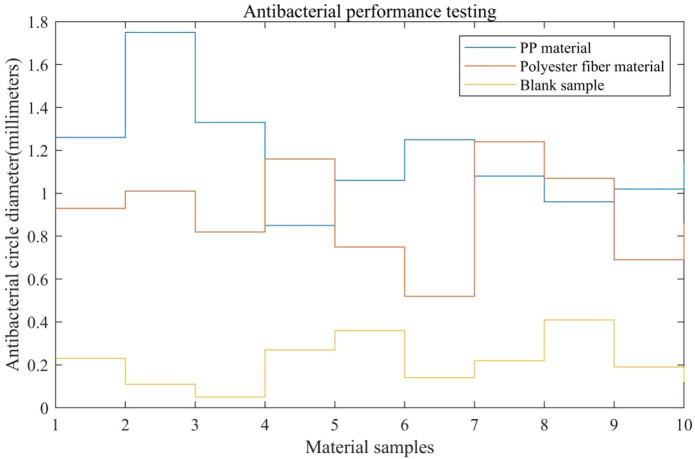
Antibacterial performance test results.

The cultivation parameters and environment of the two bacteria should be consistent. In the experiment, *Escherichia coli* and *Staphylococcus aureus* were cultured using the same medium, culture temperature, culture time, and other conditions. This can ensure that the growth state and bacterial population density of the two bacterial liquids are basically consistent. According to the parameters in Table 7, the bacterial solution was obtained, and samples of two types of equipment materials were cut into 10 cm × 10 cm (centimeter) fragments. Using a non-woven fabric as the blank sample for antibacterial testing, samples of three types of materials were placed in culture dishes and then incubated in a constant temperature incubator for 4–8 h to observe the antibacterial performance of the equipment materials. Each group tested 10 material samples and calculated the average of the 10 test results. The inhibitory effect of equipment materials was evaluated based on the diameter of the agar plate antibacterial zone as an indicator. The final result is shown in Figure 7.

In Figure 7, the horizontal axis represents the material sample used for testing, and the vertical axis represents the diameter of the antibacterial ring. Generally speaking, a bacteriostatic circle of 1 mm or more represents a good antibacterial effect of the material. From the test results, it can be seen that different materials had different levels of antibacterial performance. The maximum diameter of the antibacterial zone of the PP equipment material in this article reached 1.75 mm, and the average test diameter of the antibacterial zone was 1.17 mm. The maximum diameter of the antibacterial ring of polyester fiber material was 1.24 mm, with a testing average of approximately 0.91 mm. The maximum diameter of the antibacterial zone of the blank sample was 0.41 mm, and the average test diameter of the antibacterial zone was 0.21 mm. From the comparison results, it can be seen that the equipment material used in this article had a more significant antibacterial effect advantage, with a larger diameter of the antibacterial ring, which could effectively inhibit bacterial growth. PP materials have high chemical stability and can withstand certain chemical and physical environments, which makes them less prone to chemical changes during use, thus maintaining their good antibacterial performance.

(3) Flexibility

**FIGURE 8:**
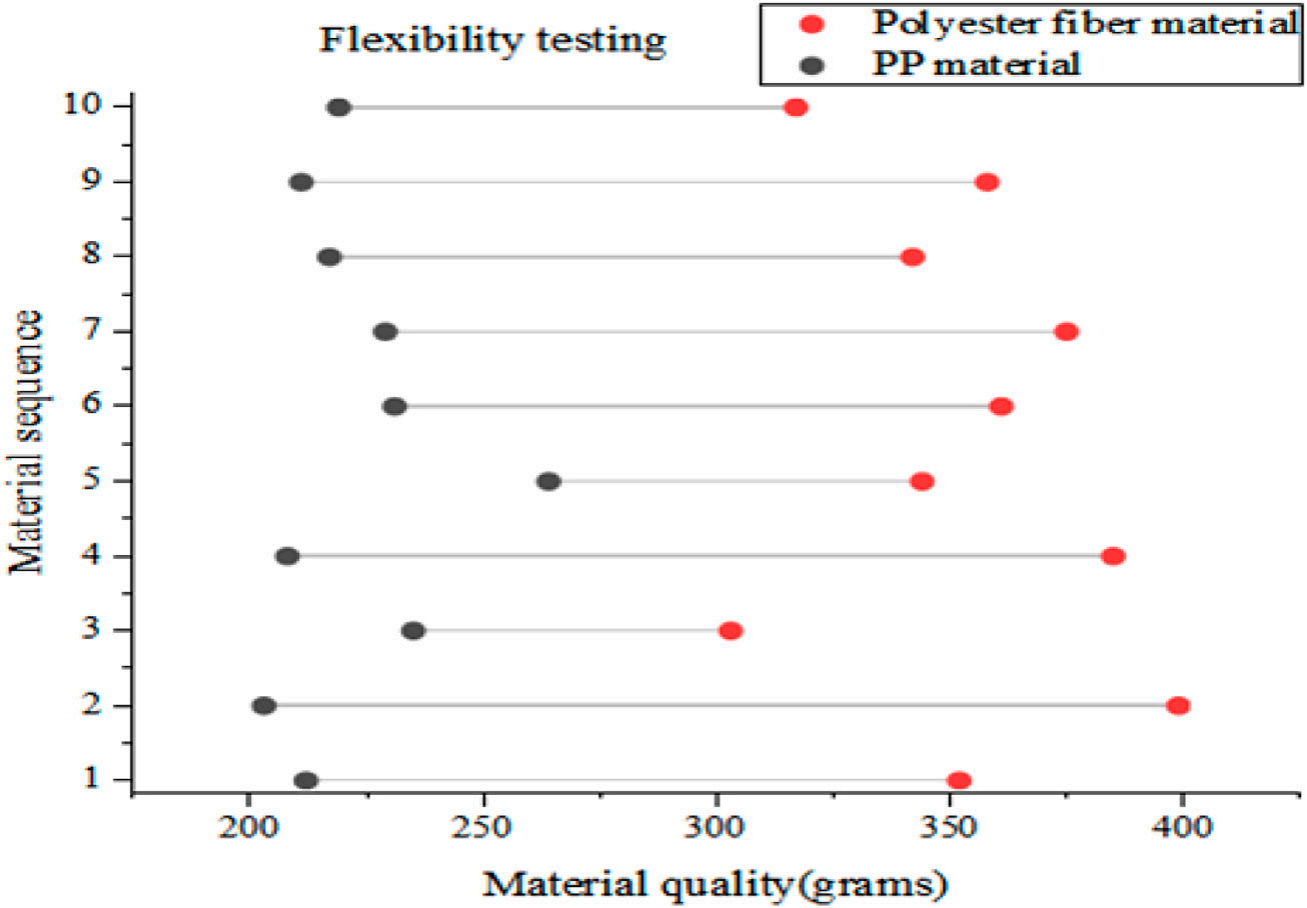
Flexibility test results.

Sports equipment with good flexibility can better adapt to the physical movements of fighting athletes, which can reduce pressure and limitations on body parts, providing better protection. To verify the flexibility of equipment materials, this article conducted flexibility tests on two types of equipment materials using equipment material quality as an indicator. Each group also tested 10 material samples, and the average of the 10 test results was taken. The final result is shown in Figure 8.

In Figure 8, the horizontal axis represents the material quality, and the vertical axis represents the material sequence. Generally speaking, lighter top equipment is suitable for high-intensity fighting during training and competition, providing athletes with better flexibility and comfort. From the flexibility test results in Figure 8, it can be seen that the equipment materials in this article had better flexibility. The average mass of sports equipment materials based on conjugated polymers in this article was 222.9 g, and the average mass of traditional polyester fiber equipment materials was 353.6 g, with a difference of over 130 g between the two. In actual fighting sports, the weight of top equipment was generally maintained within the range of 200–500 g. Within this range, the lighter the weight, the more ideal the flexibility of the equipment. From the comparison of test results, it can be seen that the equipment test results in this article have more advantages. The density of PP material is relatively small, and sports equipment based on this material is relatively lightweight, which does not cause too much burden on athletes. Moreover, the rebound characteristics of PP material enable it to stretch and contract freely with the changes in the curvature and movements of the fighting athlete’s body, thereby improving their flexibility in movement.

### 3.2 Risk of damage

Fighting exercise has a high intensity and a high risk of injury (Hammami et al., 2018). The performance of sports equipment materials plays a role in reducing the risk of injury for athletes to a certain extent. To verify the performance of fighting sports equipment developed based on conjugated polymers, this study selected six athletes from a fighting club with more than 3 years of fighting experience as the research subjects. Using the exercise data from their wearing this article’s equipment and traditional polyester fiber material equipment as experimental samples, the most common muscle and joint injury states were simulated, and the degree of reduction in injury risk was calculated.

When simulating the injury state of fighting athletes, a suitable relational model was first constructed to accurately describe the relationship between the amplitude of body changes and exercise intensity of fighting athletes during exercise. The state formula is selected (Jiebo, 2018):

$$\left\{\begin{array}{c} u=s\left(k,r\right)+d\\ v=m\left(k,r\right)+f \end{array}\right.$$ (2)

The parameters of each variable in the formula are shown in Table 8.

**TABLE 8:** Explanation of state formula variables.

Sequence	Variable	Meaning
1	u	Motion vector in motion state
2	r	Input variables
3	v	Output variables
4	d	Modeling process error
5	f	Observation error

In Table 8, the change in the value of r has a certain impact on v .

The exercise status of fighting athletes depends on the intensity of joint activity, so joint activity intensity is used to define their exercise status:

$$G=G_{0}-\frac{1}{T_{0}}\int_{0}^{4}\eta ldt$$ (3)

That is to say, when the initial value of joint activity intensity is $G_{0}$ , its instantaneous motion ability is represented as l , and it is discretized (Carey et al., 2018):

$$G_{m}=G_{m-1}-\frac{\eta l∆t}{T_{0}}$$ (4)

Furthermore, different types of damage combination motion models are selected, and the discrete results are recombined, which can be expressed as (Bulat et al., 2019):

$$v_{m}=M_{0}+M_{1}G+M_{2}/G+M_{3}\ln\left(G\right)+M_{4}\ln\left(1-G\right)$$ (5)

$v_{m}$ represents the instantaneous movement ability of a fighter’s joint in a characteristic state of motion, and the 5 constants required to maintain this state of motion are represented as $M_{0}-M_{4}$ .

In order to maintain a constant range of the relationship between the angle of change of the joint and the intensity of motion, this article uses gain matrix transformation to measure it (Hulme et al., 2019; Zhu et al., 2023):

$$P_{v}=\sum_{i=0}^{2n}\theta_{i}^{h}\left({\bar{v}}_{m}-\bar{v}\right){\left({\bar{v}}_{m}-\bar{v}\right)}^{T}$$ (6)

$$P_{u}=\sum_{i=0}^{2n}\theta_{i}^{h}\left({\bar{v}}_{m}-\bar{u}\right){\left({\bar{v}}_{m}-\bar{u}\right)}^{T}$$ (7)

$$C=P_{u}{P_{v}}^{-1}$$ (8)

According to the formulas, the relationship model between the amplitude of body changes and exercise intensity of fighting athletes can be expressed as:

$$P_{u,v}={\bar{P}}_{u,v}-CP_{v}C^{T}$$ (9)

From this, the limiting relationship between the amplitude of physical changes and exercise intensity of fighting athletes can be determined, and their sports injuries can be simulated. The exercise data of six athletes are shown in Table 9.

**TABLE 9:** Sports data of athletes.

Athlete sequence	Joint yield strength (N)	Muscle resistance (MPa)
1	433.74	392.83
2	401.58	354.11
3	376.12	327.59
4	435.71	411.35
5	440.39	421.73
6	382.16	350.29

**FIGURE 9:**
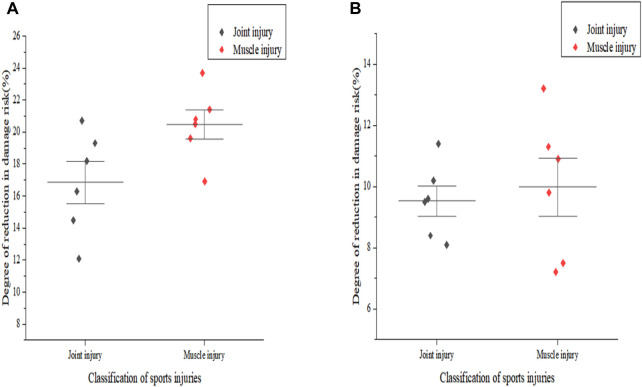
Degree of risk reduction of injury. **(A)** shows the degree of risk reduction of injury under PP material equipment. **(B)** shows the degree of risk reduction of injury under polyester fiber material equipment.

Based on the data in Table 9 and according to the formulas, the sports injury states of six athletes under two types of equipment materials were simulated. Finally, the degree of reduction in damage risk is shown in Figure 9.

In Figure 9, the horizontal axis represents the classification of sports injuries, and the vertical axis represents the degree of reduction in injury risk. From the overall comparison results, it can be seen that the equipment developed based on conjugated polymers in this article was more conducive to reducing the risk of injury for athletes. In Figure 9A, the average result of reducing the risk of joint injury for fighting athletes with material equipment in this article was about 16.9%, and the average result of reducing the risk of muscle injury was about 20.5%. In Figure 9B, the average reduction in joint injury risk for fighter athletes equipped with traditional polyester fiber materials was about 9.5%, and the average reduction in muscle injury risk was about 10.1%. From the specific injury risk test results, it can be seen that PP materials can effectively prevent joint or muscle damage in fighting athletes to a certain extent. Compared with polyester fiber materials, under PP material equipment, the average reduction in joint injury risk among athletes increased by 7.4%, and the average reduction in muscle injury risk increased by 10.4%. PP material has good mechanical properties, which can reduce impact and pressure during movement, and prevent injury and damage. In addition, its superior antibacterial performance can improve the hygiene and health of equipment, avoiding athlete injuries and infections.

## 4 Discussion

In the experimental testing of fighting sports equipment, this article verified and analyzed its effectiveness from two perspectives: equipment performance and injury risk.

(1) In equipment performance testing, this article analyzed the tensile performance, antibacterial performance, and flexibility of fighting sports equipment developed based on conjugated polymers.

In the tensile performance test, compared to traditional polyester fiber materials, the tensile strength and fracture elongation of the equipment material in this article have achieved relatively ideal results. Its unique mechanical properties advantages have provided stable support and protection for fighting athletes, improving their sports safety.

In the antibacterial performance test, the average diameter of the antibacterial ring of the PP equipment material in this article reached over 1 mm, indicating better antibacterial effect. Based on the structural characteristics of conjugated polymers, the PP material can effectively inhibit bacterial reproduction and metabolism. In fighting sports, it can not only reduce the risk of athletes’ sports injuries, but also effectively improve the comfort of equipment wearing, bringing a better sports experience for distance mobilization.

In the flexibility test, the average result of the material quality of sports equipment in this article is smaller, and its lightweight characteristic brings greater flexibility to the competition and training of fighting athletes. Moreover, based on the rebound characteristics of PP material, the sports equipment developed in this article can better fit the body of athletes and adapt to changes in their physical movements.

(2) In the injury risk testing, this article compared the degree of sports injury risk of athletes wearing two types of equipment by constructing a model of the relationship between the angle of body changes and exercise intensity of fighting athletes. From the experimental results, it can be seen that the reduction of sports injury risk of equipment materials in this article is more significant, and it can better protect far mobilization during exercise, thereby reducing the risk of joint or muscle injury in fighting athletes.

## 5 Conclusion

With the rise of fighting sports, more and more participants are joining in. As a high-intensity competitive sports event, fighting athletes also have a high risk of sports injury. Traditional sports equipment has poor antibacterial performance and flexibility, making it difficult to effectively ensure the physical safety of athletes. In order to improve the performance of sports equipment and reduce the risk of sports injuries, this article combined conjugated polymers to effectively study the development of fighting sports equipment and simulate athlete injuries. This has not only improved the mechanical properties of sports equipment to a certain extent, but also significantly enhanced its antibacterial performance and flexibility, effectively reducing the risk of athlete injuries and ensuring its safety. This article was based on the mathematical simulation research of damage detection for fighting athletes and equipment developed by conjugated polymers. Although it can provide some guidance for reducing the risk of fighting sports injuries, there are still certain limitations in the research process of this article. In the design of equipment structure, this article only developed and designed the top equipment for fighting sports, and did not consider other equipment in the research. Moreover, in the injury simulation experiment testing, the selection of sample objects in this article was not sufficient, and the impact of individual differences in athletes and differences in sports injury status on injury risk was not considered. In future research, it is necessary to consider improving the quality of research from the perspective of materials for fighting sports equipment, in order to promote the safe and healthy development of fighting sports.

## Data Availability

The original contributions presented in the study are included in the article/Supplementary Material, further inquiries can be directed to the corresponding author.
